# Translating advances in primary central nervous system lymphoma: from prognostic stratification to treatment innovation

**DOI:** 10.3389/fonc.2026.1591328

**Published:** 2026-05-07

**Authors:** Zhenjiang Pan, Jing Bao, Shepeng Wei

**Affiliations:** Department of Neurosurgery, Shanghai Shidong Hospital of Yangpu District, Shanghai, China

**Keywords:** lymphoma, high-dose methotrexate, autologous stem-cell transplantation, whole-brain radiotherapy, relapsed/refractory disease, Bruton tyrosine kinase inhibitors, CAR-T cell therapy, prognostic stratification

## Abstract

Primary central nervous system lymphoma (PCNSL) is a rare but aggressive diffuse large B-cell lymphoma confined to the CNS, characterized by unique clinical behavior and therapeutic challenges. Outcomes have improved with high-dose methotrexate (HD-MTX)-based regimens, but relapse, treatment toxicity, and age-related frailty remain major barriers. This review synthesizes advances in prognostic stratification and treatment of PCNSL. We highlight two validated clinical models (IELSG and MSKCC) and emerging genomic biomarkers that refine risk assessment. HD-MTX-based induction (MTR, R-MPV, MATRix, R-MBVP) is the standard first-line approach for fit patients, including many older adults. Consolidation with thiotepa-based high-dose chemotherapy and autologous stem-cell transplantation (HDC-ASCT) yields durable disease control in eligible patients, whereas non-myeloablative cytarabine-based chemotherapy or reduced-dose whole-brain radiotherapy remains an option for those unfit for transplant. In relapsed disease, methotrexate rechallenge benefits prior responders, while BTK- or IMiD-based regimens, CAR-T therapy, and focal or whole-brain radiotherapy are under active investigation. Maintenance with HD-MTX or targeted agents shows promise but requires validation. Although therapeutic outcomes have steadily improved, particularly with HD-MTX-based induction and HDC-ASCT consolidation, long-term survival for elderly and relapsed patients remains unsatisfactory. The integration of molecular biomarkers, neurotoxicity-sparing consolidation, and novel immunotherapies may further individualize treatment and improve the durability of remission.

## 1 Introduction

Primary central nervous system lymphoma (PCNSL) is a rare and aggressive subtype of non-Hodgkin lymphoma confined to the brain, spinal cord, cerebrospinal fluid (CSF), and ocular compartments and is biologically and clinically distinct from systemic lymphomas and other primary brain tumors. These anatomic constraints and disease-specific vulnerabilities create unique diagnostic and therapeutic challenges that require specialized clinical strategies (1, 2).

Historically, whole-brain radiotherapy (WBRT) was widely adopted as the initial treatment approach, largely extrapolated from paradigms used for gliomas and other primary brain tumors. Over the past three decades, however, the therapeutic standard has shifted substantially. High-dose methotrexate (HD-MTX)-based polychemotherapy has replaced WBRT as the backbone of first-line treatment, improving disease control while mitigating long-term neurotoxicity (1, 3).

This evolution reflects advances in the understanding of PCNSL biology and the expanding evidence base supporting risk-adapted consolidation strategies, including autologous stem-cell transplantation (ASCT), as well as the integration of targeted agents and emerging maintenance approaches (2–4). Accordingly, this review summarizes contemporary therapeutic principles and prognostic considerations in PCNSL, with an emphasis on translational advances that are shaping current and future management. Broader aspects of clinical presentation and diagnostic evaluation of PCNSL and other CNS-infiltrating lymphomas are reviewed elsewhere and provide essential clinical context (1, 2).

## 2 Search strategy

We conducted a comprehensive literature search of PubMed, Embase, and the Cochrane Library for studies published from January 2000 through February 2026, using combinations of the following keywords and Medical Subject Headings (MeSH): “primary central nervous system lymphoma,” “PCNSL,” “high-dose methotrexate,” “autologous stem-cell transplantation,” “consolidation,” “rituximab,” “ibrutinib,” “temozolomide,” “maintenance therapy,” “refractory,” “relapsed,” “neurotoxicity,” and “prognostic biomarkers”.

We prioritized randomized controlled trials, multicenter prospective studies, meta-analyses, and large real-world cohorts, but also included pivotal retrospective series that shaped current practice. Articles were screened for relevance to induction therapy, consolidation strategies, maintenance, relapse management, and long-term outcomes.

Additional references were identified by reviewing guidelines from the EHA-ESMO (2024), EANO (2023), and NCCN (2025), as well as by examining the bibliographies of key publications. Case reports and small single-center series were generally excluded unless they provided unique mechanistic or early-phase therapeutic insights.

## 3 Baseline workup

### 3.1 Histopathologic confirmation

Accurate diagnosis of PCNSL hinges on histopathologic confirmation, which remains the definitive standard. This typically requires a brain biopsy, CSF cytology, or vitrectomy. Neither neuroimaging findings—however suggestive—nor transient responses to glucocorticoids are sufficient for diagnosis; these can be misleading and must not substitute tissue-based confirmation.

Approximately 90% of PCNSL cases are classified as CD20-positive diffuse large B-cell lymphomas (DLBCLs), constituting the dominant subtype within this entity. The remainder consists of T-cell lymphomas, low-grade B-cell lymphomas, and Burkitt lymphoma, which collectively form a minority but require distinct clinical considerations. Once a diagnosis of PCNSL is established—distinguished from secondary CNS involvement by systemic lymphoma—treatment protocols generally converge for high-grade histologies. However, CD20-negative and T-cell variants necessitate rituximab-free regimens, highlighting the need for tailored therapeutic adaptation.

### 3.2 Staging and extent of disease

PCNSL exhibits a uniquely infiltrative pattern, affecting the brain parenchyma, eyes, CSF, and spinal cord. Its spread is not constrained by conventional anatomical boundaries, necessitating comprehensive staging to determine the full extent of disease. A standardized diagnostic approach includes central nervous system and ocular imaging, cerebrospinal fluid analysis, and systemic evaluation to exclude extracranial involvement. For male patients, testicular ultrasound is recommended due to the predilection for occult testicular disease (5).

Within the Ann Arbor classification for non-Hodgkin lymphoma (NHL), PCNSL is typically staged as IE, denoting isolated involvement of a single extranodal site without systemic dissemination. Although this label implies early-stage disease by traditional standards, PCNSL’s aggressive behavior and poor prognosis underscore the need for specialized staging and treatment strategies.

### 3.3 Laboratory evaluation and renal function assessment

Prior to initiating therapy for PCNSL, a comprehensive baseline laboratory workup is essential to ensure treatment safety and guide clinical decisions. Standard evaluations include the following:

Complete blood count with differential, providing insight into hematologic status.

Serum chemistries, including electrolytes, renal and hepatic function, calcium, phosphorus, and lactate dehydrogenase (LDH), which may carry prognostic significance.

Viral screening for hepatitis B (surface antigen and core antibody), hepatitis C, and human immunodeficiency virus (HIV), given the immunosuppressive nature of chemoimmunotherapy and the risk of viral reactivation.

Renal function assessment is particularly critical, as high-dose methotrexate—central to PCNSL treatment—is exclusively renally excreted. Estimated glomerular filtration rate (eGFR) should be calculated using the 2021 CKD-EPI creatinine equation. For enhanced accuracy, clinicians may consider cystatin C-based formulas, as endorsed by the National Kidney Foundation, or utilize a 24-h urine collection in select cases. Accurate renal assessment is indispensable to avoid toxicity and optimize treatment efficacy.

### 3.4 Prognostic models

In PCNSL, age and performance status remain the two most powerful predictors of overall survival (OS), a finding consistently validated across multiple studies (6–8). On the basis of these parameters, two widely used prognostic scoring systems have been developed to stratify patients and inform therapeutic expectations (6, 7).

#### 3.4.1 IELSG prognostic score

The International Extranodal Lymphoma Study Group (IELSG) model incorporates five independent prognostic variables:

Age >60 years.
Eastern Cooperative Oncology Group (ECOG) performance status >1.
Elevated serum lactate dehydrogenase (LDH).
Increased cerebrospinal fluid (CSF) protein.
Deep brain involvement (e.g., periventricular regions, basal ganglia, brainstem, or cerebellum) (7).

Patients are then categorized into three risk groups with markedly different 2-year OS outcomes:

Low risk (0–1 factors): approximately 80%.
Intermediate risk (2–3 factors): approximately 48%.
High risk (4–5 factors): approximately 15%.

#### 3.4.2 MSKCC prognostic score

The Memorial Sloan Kettering Cancer Center (MSKCC) model provides a simplified alternative that incorporates only two variables: age and Karnofsky performance status (KPS) (6). It stratifies patients into three prognostic classes:

Class 1: <50 years of age—median OS 5.2 to 8.5 years.
Class 2: ≥50 years with KPS ≥70—median OS 2.1 to 3.2 years.
Class 3: ≥50 years with KPS <70—median OS 0.9 to 1.1 years.

A comparative summary of these prognostic models is presented in **Table 1**.

**Table 1:** Prognostic scoring systems for PCNSL.

Model	Key predictors	Risk categories	Survival outcomes
IELSG	Age >60, ECOG>1, LDH, CSF protein, deep brain sites	Low (0–1), intermediate (2–3), high (4–5)	2-year OS: 80%, 48%, 15%
MSKCC	Age, KPS	Class 1 (<50), class 2 (≥50, KPS ≥70), class 3 (≥50, KPS <70)	Median OS: 5.2–8.5, 2.1–3.2, 0.9–1.1 years

Beyond conventional clinical scoring systems, genomic profiling has begun to uncover molecular determinants of outcome in PCNSL. In a recent large cohort study, Geng and colleagues demonstrated that specific genomic biomarkers were significantly associated with disease progression and overall survival, highlighting the promise of biomarker-driven risk stratification and the potential to refine prognostic models beyond age and performance status (9).

## 4 Symptom control and prophylactic considerations

PCNSL often presents with acute neurological manifestations, including altered mental status, focal deficits, headaches, seizures, and functional decline. These symptoms typically prompt a diagnostic brain biopsy and, in some cases, immediate symptom relief with glucocorticoid therapy—commonly dexamethasone at 8–16 mg/day, administered orally or intravenously in one or two divided doses (1, 2). Clinical improvement is often observed within 48–72 h, although lower doses may suffice for milder presentations.

Once induction chemotherapy is initiated, corticosteroids should be tapered to the lowest effective dose to minimize toxicity and immunosuppression. In responsive cases, steroids are typically discontinued by the second or third chemotherapy cycle.

However, the concomitant use of glucocorticoids and chemotherapy significantly increases the risk of *Pneumocystis jirovecii* pneumonia (PJP). Prophylactic measures are therefore essential. While trimethoprim–sulfamethoxazole is the standard for PJP prevention, it is often avoided in PCNSL due to its interference with high-dose methotrexate via folate antagonism. Alternative prophylactic agents should be selected strategically.

## 5 Induction chemotherapy

The treatment paradigm for PCNSL is bifurcated into two critical phases: induction therapy, aimed at achieving remission, and consolidation therapy, focused on preventing relapse. Immediate initiation of treatment following diagnosis is essential to halt neurological deterioration and prolong survival (10). HD-MTX, frequently administered in combination with rituximab and additional chemotherapeutic agents in medically fit patients, forms the cornerstone of induction. Adjustments to this regimen are necessary for patients with renal impairment, frailty, advanced age, or ocular involvement.

### 5.1 Patients with adequate fitness and kidney function

#### 5.1.1 Approach and rationale

In patients with preserved renal function (eGFR ≥30 mL/min/1.73 m^2^), HD-MTX (≥3 g/m^2^ IV) combined with adjunct chemotherapy achieves superior outcomes compared with methotrexate monotherapy or radiotherapy. Patients with eGFR <30 mL/min/1.73 m^2^ are at markedly increased risk for methotrexate-related toxicity and should be co-managed in consultation with nephrology (10–17).

Clinical trial data support the administration of four to six cycles of HD-MTX, extended beyond the point of maximal response, to enhance remission durability. In one phase II study, the combination of methotrexate and cytarabine produced a 69% objective response rate compared with 40% for methotrexate alone, albeit with greater toxicity (overall survival hazard ratio 0.65, 95% CI 0.38–1.13) (14).

The benefit of adding rituximab to CD20-positive regimens remains uncertain. The HOVON-105/ALLG-NHL-24 phase III trial showed no statistically significant survival advantage after a median follow-up of 6.9 years (18, 19). By contrast, the IELSG32 phase II trial reported that methotrexate, cytarabine, and rituximab improved 7-year overall survival compared with methotrexate and cytarabine alone (37% *vs*. 21%; hazard ratio 0.64; 95% CI 0.41–0.99) (20). Rituximab may therefore be omitted in patients at high risk for severe immunosuppression.

Given its critical role in achieving therapeutic central nervous system penetration, HD-MTX requires aggressive hydration and frequent renal monitoring throughout the treatment (17).

#### 5.1.2 Specific regimens

There remains a paucity of head-to-head trials directly comparing induction regimens for PCNSL. Consequently, clinical decisions are often guided by institutional experience, patient characteristics, and physician judgment. Across cooperative group studies, various methotrexate-based multi-agent protocols have demonstrated broadly comparable complete response (CR) and overall response (OR) rates, though direct comparisons are hampered by study heterogeneity.

In the absence of a definitive standard, four induction regimens—refined in single-arm and randomized phase II studies—have emerged as leading candidates for first-line therapy in PCNSL:

- MTR: high-dose methotrexate (8 g/m^2^), temozolomide, and rituximab administered in four 28-day cycles (21). In the multicenter Alliance 50202 trial, 44 newly diagnosed patients (median age 61; range 12–76) received MTR induction followed by non-myeloablative etoposide and cytarabine (EA) consolidation (10). CR and OR rates were 66% and 77%, respectively, with 2-year progression-free survival (PFS) of 57% and a 4-year OS estimate of 65%. In the randomized Alliance 51101 trial, MTR with one cycle of high-dose cytarabine (MTR-A) achieved a 50% CR rate prior to consolidation with either EA or high-dose chemotherapy and autologous stem-cell transplantation (HDC-ASCT) (21).
- R-MPV: rituximab, methotrexate (3.5 g/m^2^), procarbazine, and vincristine given every 14 days for 5–7 cycles (12). Developed at Memorial Sloan Kettering Cancer Center, this regimen was followed by reduced-dose whole-brain radiotherapy and cytarabine, yielding a 78% CR rate and a 2-year PFS of 57% (12). Subsequent multicenter studies reported OR rates of 79%–83%, and the addition of cytarabine (R-MPV-A) achieved a 2-year PFS of 54% (22, 23).
- MATRix: methotrexate (3.5 g/m^2^), cytarabine (2 g/m^2^ every 12 h × 4 doses), thiotepa, and rituximab. In the IELSG32 randomized phase II trial involving 227 patients aged 18–70, MATRix achieved CR and OR rates of 49% and 87%, respectively, with a 7-year OS of 56% after a median follow-up of 7.3 years (13, 20, 24).
- R-MBVP: rituximab, methotrexate (3 g/m^2^), carmustine, teniposide (or etoposide), and prednisolone, typically for two 28-day cycles, often followed by cytarabine (2 g/m^2^ every 12 h × 4 doses; R-MBVP-A) (25). In the PRECIS and HOVON105 studies, which primarily included patients ≤60 years of age, CR rates ranged from 43% to 45% and OR from 70% to 82% (18, 25). The principal features and outcomes of these regimens are summarized in **Table 2**.

**Table 2:** Common induction chemotherapy regimens.

Regimen	Components	Methotrexate dose	Cycles	CR rate	OR rate	PFS/OS
MTR	Methotrexate, temozolomide, rituximab	8 g/m^2^	4	66%	77%	2-year PFS: 57%
R-MPV	rituximab, methotrexate, procarbazine, vincristine	3.5 g/m^2^	5–7	78%	79%–83%	2-year PFS: 54%–57%
MATRix	Methotrexate, Cytarabine, thiotepa, rituximab	3.5 g/m^2^		49%	87%	7-year OS: 56%
R-MBVP	Rituximab, methotrexate, carmustine, etoposide/teniposide, prednisolone	3 g/m^2^	2	43%–45%	70%–82%	Varies by trial

### 5.2 Patients with low performance status or organ dysfunction

Patients with PCNSL often present with poor performance status at diagnosis. However, neurologic dysfunction—particularly when driven by mass effect from intracranial lesions—should not automatically preclude induction therapy, since effective treatment can reverse functional decline. Renal impairment likewise does not represent an absolute contraindication to methotrexate-based induction; with careful monitoring, dose adjustment, and contingency planning for renal toxicity, treatment can be delivered safely.

By contrast, high-dose methotrexate presents significant challenges in patients with congestive heart failure and reduced ejection fraction due to risks of pulmonary edema and fluid overload. These risks are further amplified in the presence of third-space fluid accumulation (e.g., pleural effusions, ascites), which can entrap methotrexate. Standard protocols typically require 2.5–3.5 L/m^2^ of intravenous hydration per day, initiated 4–12 h before methotrexate administration and continued for 2–4 days afterward. In patients with heart failure, eligibility for such hydration should be carefully assessed by baseline echocardiography and monitored through 24-h fluid balance measurements.

For patients deemed ineligible for high-dose methotrexate, treatment options are more limited. Those with substantial comorbidities or frailty may begin with single-agent methotrexate, with or without rituximab, and escalate therapy if functional status improves. Alternatively, temozolomide—alone or in combination with rituximab—serves as a palliative approach for patients unable to tolerate methotrexate or cytarabine (26, 27). Radiotherapy also remains valuable, particularly in frail patients requiring symptom relief. Although novel agents such as ibrutinib appear promising, they currently lack robust evidence as monotherapy and should be considered investigational outside of clinical trials.

**Figure 1:**
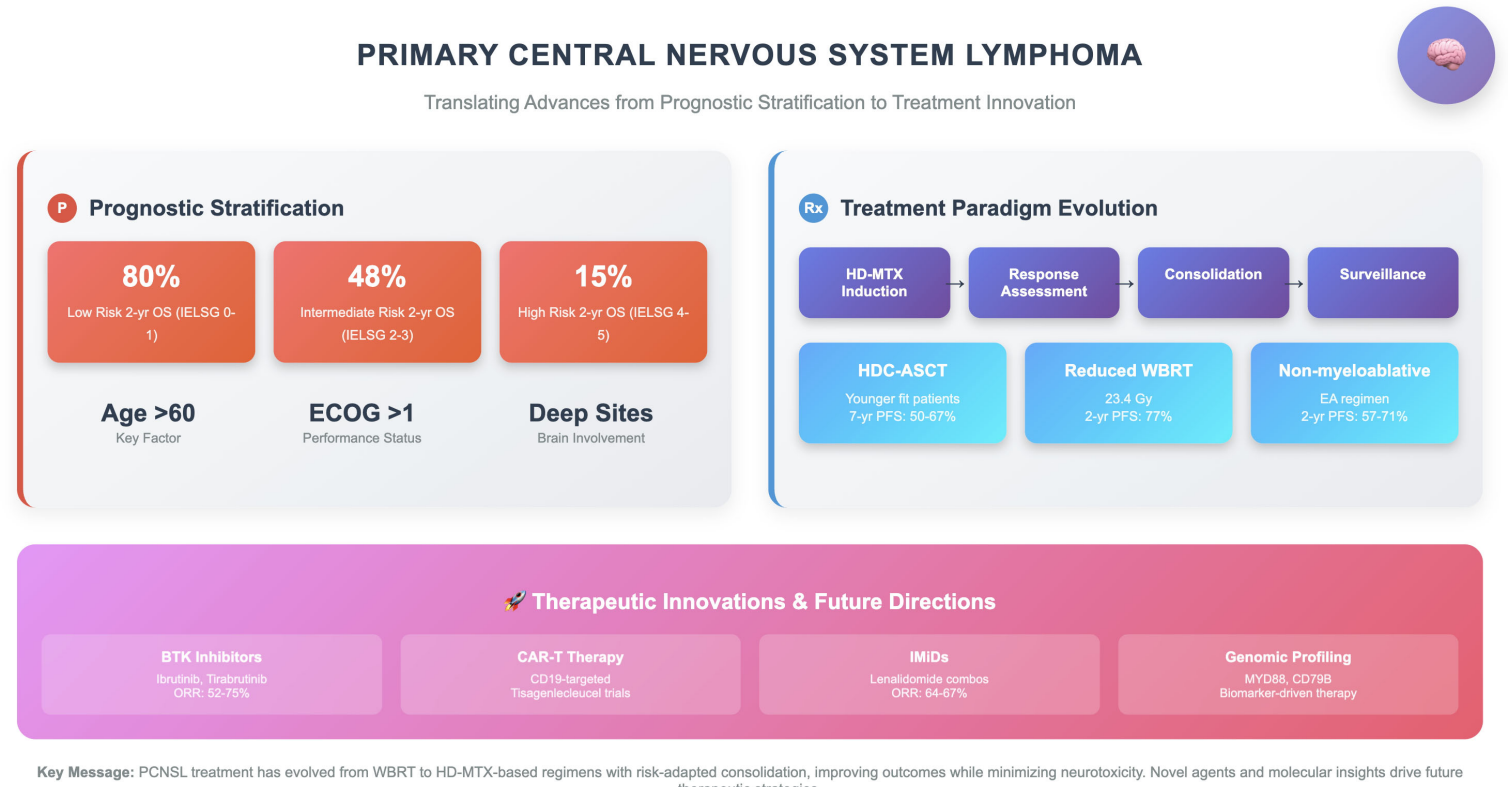
Integrated prognostic and therapeutic pathways in primary CNS lymphoma.

A practical treatment pathway for PCNSL, integrating induction, consolidation, and salvage strategies, is illustrated in **Figure 1**.

### 5.3 Older adults

Clinical trials and retrospective analyses have dispelled the outdated notion that advanced age alone should preclude the use of HD-MTX in patients with PCNSL. Evidence indicates that patients aged 65 years or older—and even those over 80—can safely receive HD-MTX, provided renal function and performance status are adequate (28–31). For older but functionally fit individuals, methotrexate-based regimens incorporating oral alkylating agents, such as MTR or R-MPV, have shown favorable tolerability and efficacy. In a French phase II trial enrolling 98 patients aged 60 years or older (median age 72), MPV-A was compared with a methotrexate–temozolomide regimen (without rituximab). Both approaches produced comparable progression-free and overall survival outcomes, with acceptable toxicity profiles (32).

In contrast, intensive consolidative strategies such as high-dose cytarabine are poorly tolerated in elderly patients owing to heightened risks of treatment-related morbidity and mortality. A meta-analysis of 783 patients with PCNSL aged 60 years or older showed that methotrexate-based regimens with oral alkylators achieved overall survival similar to that of more aggressive combinations (hazard ratio, 1.39; 95% CI 0.90–2.15), without excessive toxicity (28).

Beyond conventional HD-MTX-based approaches, novel combinations are being explored to improve induction efficacy. In a prospective multicenter phase II trial, Gao and colleagues demonstrated that the addition of ibrutinib and temozolomide to high-dose methotrexate achieved encouraging responses with manageable toxicity in newly diagnosed PCNSL, underscoring the feasibility of incorporating targeted and alkylating agents into frontline induction (33).

### 5.4 Patients with eye involvement

Ocular involvement in PCNSL may occur concurrently with brain or CSF disease or present independently as primary vitreoretinal lymphoma (PVRL). In cases with concurrent CNS and ocular disease, standard induction and consolidation regimens are typically sufficient, with retrospective data suggesting that ocular involvement does not independently worsen prognosis (8, 34). In contrast, isolated PVRL poses a unique therapeutic challenge due to the lack of standardized treatment protocols. Local modalities—such as intravitreal methotrexate or rituximab injections and ocular radiation—can achieve direct control of intraocular disease, while systemic high-dose methotrexate offers CNS penetration. However, relapse in both compartments remains common despite aggressive regimens, including high-dose chemotherapy, autologous transplantation, or WBRT (11, 35, 36).

Management of PVRL typically involves a multidisciplinary team, integrating neuro-oncology, ophthalmology, and radiation oncology expertise. For younger, fit patients, the preferred strategy combines local therapy with systemic high-dose methotrexate to prolong PFS, while avoiding intensive consolidative approaches such as WBRT or high-dose chemotherapy in frailer individuals (35). For selected robust patients, more aggressive regimens may incorporate systemic methotrexate, intravitreal agents, ocular radiation, and even HDC-ASCT (35).

### 5.5 Limited role of intrathecal therapy

Intrathecal methotrexate, once a mainstay in the induction strategy for PCNSL, has now largely fallen out of favor—even in cases marked by CSF dissemination. The decisive blow came from studies that dismantled earlier assumptions, demonstrating that intravenous (IV) methotrexate, when administered at doses exceeding 3 g/m^2^, achieves therapeutic micromolar concentrations within the CSF (37, 38). Given this systemic potency, the risks and complications associated with intrathecal delivery—particularly through an Ommaya reservoir—are no longer justified. Repeated reservoir access carries a tangible risk of infection and a cascade of procedure-related complications (39, 40). With IV high-dose methotrexate effectively saturating the CSF, intrathecal therapy has been relegated to the shadows.

## 6 Efficacy assessment

For patients engaged in the high-stakes battle against PCNSL, the induction phase requires vigilant and ongoing assessment. Frequent clinical evaluations help track neurologic recovery, with many patients demonstrating marked improvement within one or two cycles of high-dose methotrexate—occasionally within days of the initial infusion.

Contrast-enhanced brain MRI serves as the principal imaging modality to monitor treatment response, typically performed after every two to four methotrexate doses. The International PCNSL Collaborative Group (IPCG) provides standardized response criteria to guide interpretation and clinical decision-making (5). For patients with baseline involvement of the eyes, spinal cord, or CSF, serial ophthalmologic exams, spine MRI, and CSF cytology remain essential adjuncts for comprehensive disease monitoring.

Emerging imaging technologies, particularly coregistered fluorodeoxyglucose positron emission tomography (FDG-PET)/MRI, offer promise in refining response evaluation and detecting early signs of disease progression, especially in cases with ambiguous MRI findings. However, its role remains investigational, pending validation in prospective clinical trials (41).

### 6.1 Patients with responsive disease

In the therapeutic course of PCNSL, achieving a CR during induction marks the gateway to consolidation. Up to eight cycles of high-dose methotrexate serve as the backbone of induction, with patients reaching CR within the first four doses typically receiving an additional one to two cycles—amounting to two to four total doses—tailored to renal function and overall tolerance, before proceeding to consolidation when clinically appropriate.

For those attaining a partial response (PR), some protocols advocate transitioning directly to consolidation upon initial signs of response, minimizing further methotrexate exposure. However, when patients remain in PR despite completing eight doses of methotrexate and demonstrate preserved functional status and radiographic improvement, an additional cycle of high-dose cytarabine (2 g/m^2^ every 12 h for four doses) is often employed as salvage intensification. If stability or further improvement is confirmed post-cytarabine, the patient may proceed to consolidation.

In select transplant-eligible patients who remain in PR after induction, direct transition to HDC-ASCT may be pursued, bypassing further cytotoxic escalation during induction.

### 6.2 Patients with primary refractory disease

Primary refractory disease in PCNSL represents a formidable clinical challenge. This diagnosis is defined in patients who fail to respond to at least two doses of high-dose methotrexate administered at central nervous system-penetrating levels (≥1 g/m^2^, with ≥3.5 g/m^2^ considered optimal, accounting for dose reductions). Even cases classified as stable disease under these conditions are considered refractory, a particularly concerning scenario given the usual chemosensitivity of PCNSL to methotrexate-based regimens.

No universally accepted second-line standard exists for this population. Enrollment in clinical trials exploring novel therapeutic agents remains the preferred strategy. In the absence of trial access, high-dose cytarabine—either as monotherapy or in combination with etoposide—remains a commonly used salvage option for patients unresponsive to methotrexate (42). Patients achieving a response may subsequently proceed to consolidation therapy.

For patients who fail to respond to cytarabine-based salvage, the prognosis is dismal. Focal radiotherapy may provide temporary disease control and symptom relief, occasionally serving as a bridge to additional salvage attempts in selected individuals. The use of single-agent ibrutinib in this setting has shown only modest and often short-lived activity, underscoring the pressing need for more effective strategies.

Among salvage regimens, polychemotherapy remains a viable option in selected patients. In a large multicenter study from the LOC network, Pérol and colleagues demonstrated that ICE (ifosfamide, carboplatin, and etoposide) produced clinically meaningful responses in relapsed or refractory PCNSL, supporting its continued role as an effective cytotoxic backbone in the salvage setting (43).

## 7 Consolidation treatment

Following successful induction therapy for PCNSL, three principal modalities have emerged as leading consolidation strategies: HDC-ASCT, non-myeloablative chemotherapy, and reduced-dose WBRT. Each has demonstrated efficacy in prospective studies, including select randomized trials, and represents a viable pathway to sustained disease control.

However, no definitive comparative trials have established superiority among these options. Their differing toxicity profiles further complicate clinical decision-making. HDC-ASCT, while potentially curative, is often limited to younger or physiologically fit individuals due to its intensity. Non-myeloablative chemotherapy offers a less toxic alternative, albeit with less robust long-term data. Reduced-dose WBRT, once a mainstay, now balances concerns about late neurotoxicity against its consolidation potential.

In clinical practice, these strategies remain largely patient-tailored, with age, performance status, neurocognitive reserve, and prior treatment response shaping the final choice. For many older or medically frail patients, however, all three options may be impractical—underscoring the urgent need for safer and more effective consolidation approaches.

### 7.1 Consolidation strategy selection

The selection of consolidation therapy in PCNSL follows a risk-adapted framework:

For younger, fit patients who achieve a CR or a favorable PR after induction, thiotepa-based HDC-ASCT is the preferred approach. Early referral for transplant evaluation—ideally midway through induction—helps prevent delays in transitioning to consolidation. If the interval between induction completion and transplant exceeds 3–4 weeks, an interim cycle of high-dose cytarabine may be administered to maintain disease control.

For patients with chemosensitive disease but deemed ineligible for HDC-ASCT due to advanced age or significant comorbidities, non-myeloablative chemotherapy serves as an alternative. Common regimens include high-dose cytarabine or the etoposide–cytarabine (EA) combination. Despite reduced intensity, these regimens carry notable hematologic toxicity and require individualized risk–benefit assessment. In patients over 75 years of age, their use is uncommon due to limited tolerance.

Reduced-dose WBRT remains a fallback option for patients with responsive disease who are unsuitable for chemotherapy-based consolidation due to organ dysfunction or personal preference. Given the risk of delayed neurotoxicity—which increases with age—WBRT is typically reserved for individuals under 65 years old. **Table 3** provides a comparative summary of these consolidation modalities, stratified by patient fitness and treatment goals.

**Table 3:** Consolidation therapy options.

Option	Target population	Key agents	Efficacy	Toxicity risks
HDC-ASCT	Young, fit, responsive	Thiotepa-based (TBC, TT-BCNU)	7-year PFS: 50%–67%	Early mortality, infection
Non-myeloablative chemotherapy	ASCT eligible	Cytarabine ± etoposide	2-year PFS: 57%–71%	Hematologic toxicity
Reduced-dose WBRT	Responsive, chemo-ineligible	23.4 Gy	2-year PFS: 77%	Delayed neurotoxicity

#### 7.1.1 High-dose chemotherapy and autologous stem cell transplantation

HDC-ASCT represents a highly effective consolidation strategy for select younger and physiologically fit patients with chemotherapy-responsive PCNSL.

The optimal conditioning regimen, however, remains undefined. Accumulating evidence favors thiotepa-containing protocols—such as TBC (thiotepa, busulfan, cyclophosphamide) and TT-BCNU (thiotepa and carmustine)—over regimens more commonly employed in systemic DLBCL, including BEAM (carmustine, etoposide, cytarabine, melphalan) and CBV (cyclophosphamide, etoposide, carmustine) (44–46). A retrospective analysis of the Center for International Blood and Marrow Transplant Research registry confirmed superior survival outcomes with thiotepa-based conditioning (47).

Among the thiotepa-based options, TBC appears to confer a modest survival advantage over TT-BCNU, albeit at the cost of increased toxicity and non-relapse mortality, underscoring the need for careful patient selection.

When compared with WBRT, HDC-ASCT demonstrates equivalent disease control but avoids the long-term neurocognitive sequelae associated with radiation. While HDC-ASCT carries a higher risk of early treatment-related mortality, WBRT is more frequently associated with delayed complications, including vascular events and cognitive decline, which may impact long-term survival and quality of life.

Two randomized trials have directly compared HDC-ASCT and WBRT as consolidation strategies in PCNSL:

- IELSG32 trial – In this two-stage, phase 2 International Extranodal Lymphoma Study Group-32 (IELSG32) trial, 118 of 227 enrolled patients (aged 18–70 years) with responsive or stable disease after induction were randomized to receive HDC-ASCT or WBRT (24). After a median follow-up of 30 months, the 2-year PFS was comparable between groups [69% for HDC-ASCT *vs*. 88% for WBRT; hazard ratio (HR) 1.50, 95% confidence interval (CI) 0.83–2.71]. Two treatment-related deaths from infection occurred in the HDC-ASCT arm. At a median follow-up of 88 months, 7-year PFS remained similar (50% *vs*. 55%), as did 7-year OS (57% *vs*. 63%) for HDC-ASCT and WBRT, respectively (20).
- Importantly, prospective neurocognitive and quality-of-life assessments revealed a marked decline in attention and executive function among WBRT recipients, while patients in the HDC-ASCT group exhibited relative cognitive preservation and improvement over time (20, 24).
- PRECIS trial – This phase 2 randomized trial enrolled 140 newly diagnosed PCNSL patients aged ≤60 years and randomized them to receive consolidation with either HDC-ASCT or WBRT (40 Gy) following induction with high-dose methotrexate and cytarabine (25). At a median follow-up of 30 months, the 2-year event-free survival (EFS) slightly favored HDC-ASCT (70%, 95% CI 59–82) over WBRT (58%, 95% CI 47–71), though the difference was not statistically significant. The 2-year OS was similar between arms (66% *vs*. 75%), but early treatment-related mortality was higher with HDC-ASCT (five deaths *vs*. one).

By 8.2 years of follow-up, OS remained comparable (54% *vs*. 58%) (48). However, 13 patients in the HDC-ASCT arm experienced progression during induction and did not undergo consolidation, compared to only 5 in the WBRT arm. Among those who completed HDC-ASCT per protocol, the 8-year EFS was 67%, compared to 39% in the per-protocol WBRT group. Cognitive function remained stable or improved in the HDC-ASCT cohort at 30 months, whereas WBRT was associated with declines in attention and executive function. Long-term neurotoxicity was significantly greater in the WBRT arm, with 64% experiencing cognitive decline, 52% reporting balance disturbances, and four late deaths attributed to radiation toxicity (25, 48).

Nevertheless, the long-term neurocognitive risks of HDC-ASCT versus reduced-dose WBRT remain uncertain. Differences in induction regimens, WBRT dosing, and neurocognitive assessment tools across studies introduce variability. A longitudinal cohort study following PCNSL patients for up to 5 years after consolidation with either HDC-ASCT or reduced-dose WBRT reported initial cognitive improvement over the first 3 years, followed by a gradual decline in both groups (49).

#### 7.1.2 Non-myeloablative chemotherapy as consolidation

For patients with PCNSL who achieve a response to induction but are ineligible for HDC-ASCT owing to advanced age or comorbidities, non-myeloablative chemotherapy represents a pragmatic consolidation strategy, particularly in those ≤75 years with adequate performance status (10). Commonly used regimens include high-dose cytarabine alone or in combination with etoposide (EA), though hematologic toxicity remains a significant limitation in older adults.

In the CALGB 50202 trial, 44 newly diagnosed patients (median age 61, range 12–76) received MTR induction (methotrexate 8 g/m^2^, rituximab, temozolomide). Among the 29 patients who achieved a CR, consolidation with EA (etoposide 5 mg/kg every 12 h × 8 doses; cytarabine 2 g/m^2^ every 12 h × 8 doses) yielded a 2-year PFS of 57%, with median OS not reached; one treatment-related death occurred (10).

Subsequently, the CALGB 51101 randomized phase II trial compared EA with HDC-ASCT in 113 patients aged ≤75 years following MTR-A induction. After a median follow-up of 4.1 years, HDC-ASCT achieved superior median PFS (HR 0.51, 95% CI 0.29–0.90) (50). During induction, disease progression or death occurred in 28% of patients in the EA arm versus 11% in the HDC-ASCT arm. Among the 70 patients who proceeded to consolidation, the HDC-ASCT group achieved a 2-year PFS of 86% compared with 71% for EA (HR 0.58, 95% CI 0.25–1.36). Median OS was not reached in either group, with 2-year OS exceeding 90% (50).

Given the substantial hematologic toxicity of EA—particularly in older or frail patients—attenuated cytarabine-only regimens (e.g., 2 g/m^2^ every 12 h × 4 or 3 g/m^2^ every 24 h × 2) have been explored as more tolerable alternatives.

Complementing these trial data, real-world institutional experience has further reinforced the feasibility and effectiveness of MTR induction followed by consolidation, as reported by Dave and colleagues (51).

#### 7.1.3 Reduced-dose whole-brain radiotherapy as consolidation

WBRT remains a consolidation option for patients with newly diagnosed PCNSL, particularly those unfit for high-dose chemotherapy. However, its application is tempered by the risk of delayed neurotoxicity, prompting exploration of reduced-dose strategies (e.g., 23.4 Gy) aimed at preserving cognitive function while maintaining disease control (22, 23, 25, 52).

A single-arm study evaluated reduced-dose WBRT (23.4 Gy) plus cytarabine in 31 patients who achieved CR after R-MPV induction. This regimen yielded a 2-year PFS of 77% and a 3-year OS of 87%, with preserved cognitive function. In contrast, non-CR patients received standard-dose WBRT (45 Gy) (22). The NRG Oncology phase 2 trial further investigated the impact of reduced-dose WBRT. Patients received R-MPV-A induction and were randomized to either observation or consolidation with 23.4 Gy WBRT. The WBRT arm showed improved 2-year PFS, with early signs of neurocognitive preservation, although long-term toxicity and OS data remain pending (23).

Conversely, a large German cohort study of 551 patients compared methotrexate-based induction followed by 45 Gy WBRT (1.5 Gy × 30 fractions) versus methotrexate with response-adapted chemotherapy (cytarabine for non-CR, observation for CR). After a median follow-up of 50 months, WBRT significantly improved PFS (HR 0.79, 95% CI 0.63–0.99) but did not affect OS (HR 1.01, 95% CI 0.79–1.30). Among 80 survivors assessed, neurotoxicity rates were notably higher in the WBRT group (49% *vs*. 26%) (53).

The combination of high-dose methotrexate with WBRT increases the risk of irreversible neurotoxicity, particularly in patients over 60 years. Affected domains include psychomotor speed, attention, executive function, and memory, with MRI frequently revealing diffuse white matter changes and cerebral atrophy. However, the correlation between imaging findings and functional impairment remains inconsistent (54–56). These concerns have fueled the search for alternative consolidation strategies with reduced neurocognitive burden.

### 7.2 Consolidation in older or frail patients

For older adults, those with impaired performance status, or patients teetering at the brink of neurotoxicity, a safe yet effective consolidation strategy remains elusive—a tantalizing mirage on the treatment horizon. In these cases, management becomes a bespoke gamble, tailored to each patient’s physiologic reserve and therapeutic history. Whether gentler post-induction strategies outperform observation remains uncertain; some patients in CR after high-dose methotrexate maintain durable remissions for months or even years without further intervention.

Outside clinical trial settings, a watch-and-wait approach often prevails—particularly for patients achieving CR following induction. For those deemed fit, some experts favor a low-intensity maintenance phase with monthly high-dose methotrexate infusions for up to 1 year (57). Oral agents such as temozolomide, procarbazine, and ibrutinib have also been explored, but their efficacy in this context remains largely anecdotal.

Maintenance therapy with these agents has demonstrated feasibility and tolerability in small, non-randomized trials involving older or frail PCNSL survivors, including those aged ≥65 years (58–61). However, definitive evidence remains lacking. A phase 3 randomized trial conducted by the Japan Clinical Oncology Group enrolled 122 patients to receive high-dose methotrexate induction followed by either WBRT alone or WBRT plus temozolomide (concurrent and adjuvant) (62). Interim analysis showed no survival benefit with temozolomide; in fact, the 2-year OS was lower in the temozolomide arm (71% *vs*. 87%; HR 2.18, 95% CI 0.95–4.98), leading to early termination of the trial for futility.

Hope, however, remains on the horizon. Ongoing studies are investigating the use of immunomodulatory agents and immune checkpoint inhibitors in the maintenance setting—novel contenders in an arena still searching for a champion (63).

Recent real-world data from the Mayo Clinic compared outcomes across different post-induction strategies and suggested that maintenance high-dose methotrexate may serve as a time-limited treatment option for patients achieving remission, thereby providing evidence to bridge the gap between consolidation and observation (64).

## 8 Surveillance and long-term follow-up

Following treatment, patients with PCNSL face a persistent risk of relapse, necessitating vigilant surveillance. Relapsed disease can progress swiftly, often causing irreversible neurologic deterioration. To detect recurrence early, the International Primary CNS Lymphoma Collaborative Group (IPCG) recommends contrast-enhanced brain MRI every 3 months for the first 2 years, every 6 months for the next 3 years, and annually thereafter—extending up to 10 years (5), or indefinitely in selected cases (57). Notably, late relapses beyond a decade have been documented (65), and in one cohort of 256 patients, 25% of relapses were asymptomatic and detected solely through surveillance imaging (66).

In parallel, long-term survivors are at risk for neurocognitive decline due to treatment-related toxicity. As such, the IPCG endorses serial monitoring of cognitive function and quality of life at each follow-up visit (54). This includes clinical history, targeted neurologic examinations, and formal neuropsychological testing when cognitive impairment is suspected, facilitating early intervention and longitudinal tracking of cognitive trajectories.

## 9 Recurrent/refractory condition

In the unforgiving arena of PCNSL, approximately 25% of patients exhibit primary resistance to initial HD-MTX-based therapy, while nearly 50% ultimately relapse after an initial response. For those facing relapsed or refractory disease, prognosis remains dismal, and no universally accepted salvage strategy has emerged. Randomized trials are conspicuously absent, and therapeutic decisions are highly individualized—contingent on patient age, performance status, neurological function, comorbidities, site of relapse, prior treatments, and the duration of the preceding remission. In this landscape, enrollment in clinical trials remains a top priority whenever available.

### 9.1 Methotrexate sensitivity and prolonged remission

For patients who previously responded favorably to HD-MTX and achieved a prolonged remission—typically defined as 12 to 24 months or longer—rechallenge with HD-MTX remains a rational and potentially rewarding strategy. Preferred regimens include HD-MTX in combination with rituximab, although other MTX-based combinations are also considered.

Retrospective studies support this approach: in first-relapse PCNSL, HD-MTX rechallenge yields overall response rates of 80%–90%, with median overall survival extending from 40 to 60 months (67–69). For patients who have not yet undergone HDC-ASCT, consolidation with HDC-ASCT after re-induction may be appropriate, extrapolating from successes in both newly diagnosed and refractory PCNSL populations (42, 45, 46, 70). **Table 4** summarizes the efficacy of available salvage strategies in recurrent and refractory PCNSL.

**Table 4:** Relapsed/refractory treatment outcomes.

Treatment	Eligibility	OR rate	Median PFS	Median OS
Methotrexate rechallenge	Prior responders, >12–24 months remission	80%–90%	–	40–60 months
Thiotepa-based HDC-ASCT	Methotrexate-refractory, fit	63%–75%	11.6–41 mo	18.3–59 months
lbrutinib monotherapy	All relapsed/refractory	52%–75%	2-4.8 mo	–
Radiation (WBRT)	Rapid neurologic decline	74%–79%	–	10–16 months

### 9.2 High-dose chemotherapy and ASCT in methotrexate-refractory PCNSL

#### 9.2.1 Candidates for high-dose chemotherapy

For fit patients with methotrexate-refractory PCNSL at first relapse, thiotepa-based HDC-ASCT represents a promising salvage strategy (42, 70).

A French phase 2 study evaluated this approach in 43 patients with relapsed or refractory PCNSL (median age 52; range 23–65 years), utilizing etoposide-cytarabine (EA) induction followed by thiotepa, busulfan, and cyclophosphamide (TBC) conditioning prior to ASCT (42). Despite three induction-related deaths, outcomes were favorable among the 27 patients who successfully completed ASCT, achieving a median PFS of 41 months and OS of 59 months. For the entire cohort, median PFS and OS were 11.6 and 18.3 months, respectively.

A separate German study enrolled 39 patients aged ≤65 years (median 57), who received two cycles of rituximab, high-dose cytarabine, and thiotepa, with stem cell harvest performed mid-treatment (70). Thirty-two patients proceeded to ASCT, with four treatment-related deaths reported. After a median follow-up of 45 months, the cohort demonstrated a median PFS of 12.4 months and a 2-year OS of 56.4%, while median OS remained unreached.

Evidence supporting allogeneic hematopoietic stem-cell transplantation (allo-HSCT) in relapsed/refractory PCNSL remains extremely limited. Available data are largely confined to case reports and very small retrospective series (71, 72). In a small retrospective cohort, long-term lymphoma-free survival was observed in some selected patients after allo-HSCT, although treatment-related complications, including graft-versus-host disease, remained important concerns (71). Therefore, allo-HSCT should not be regarded as a standard salvage strategy in PCNSL and may be considered only in highly selected cases at experienced centers, particularly when autologous transplantation is not feasible (71, 72).

#### 9.2.2 All other patients

For patients with relapsed/refractory PCNSL who are not candidates for high-dose chemotherapy followed by autologous stem-cell transplantation, therapeutic options remain limited and durable disease control is uncommon. Management may include single-agent or combination systemic therapy, radiotherapy, and participation in clinical trials evaluating novel agents. No single salvage approach has emerged as a universal standard; therefore, treatment should be individualized based on prior therapy, duration of remission, performance status, neurologic disease burden, comorbidities, and treatment goals.

Systemic therapies: current landscape and emerging options.

① Chemotherapy: limited durability.

Several chemotherapeutic agents, including pemetrexed, topotecan, rituximab, and temozolomide, have demonstrated modest activity in prospective trials for relapsed or refractory PCNSL, achieving objective responses in select patients (26, 73–77). However, PFS remains limited, typically ranging from 2 to 6 months, highlighting the transient efficacy of these regimens.

② BTK inhibitors: encouraging activity, modest durability.

Bruton’s tyrosine kinase (BTK) inhibitors, particularly ibrutinib and tirabrutinib, have shown promising single-agent activity in early-phase trials but with limited durability of response.

A phase 1 dose-escalation trial of ibrutinib (560–840 mg daily) in relapsed/refractory PCNSL and secondary CNS lymphoma reported a 75% objective response rate (ORR), with a median PFS of 4.6 months (78).

In a multicenter French phase 2 study involving 52 patients (including vitreoretinal lymphoma), daily ibrutinib (560 mg) achieved a 52% ORR at 2 months, with a median PFS of 4.8 months. Among patients without vitreoretinal involvement, median PFS dropped to 2 months (79).

Tirabrutinib, a second-generation BTK inhibitor, demonstrated an ORR of 63.4% and a median PFS of 2.9 months in a Japanese phase 1/2 study (80), leading to regulatory approval in Japan for relapsed/refractory PCNSL in March 2020 (81). A phase 2 trial is ongoing in the United States.

BTK inhibitor-based combinations have demonstrated improved outcomes. In a phase 1 study, a regimen comprising ibrutinib, temozolomide, etoposide, liposomal doxorubicin, dexamethasone, rituximab (TEDDI-R), and intrathecal cytarabine yielded a 93% ORR (predominantly complete responses) and a median PFS of 15.5 months among 14 evaluable patients (5 newly diagnosed, 13 relapsed/refractory) (82). Another phase 1 study combining ibrutinib with high-dose methotrexate and rituximab reported an ORR of 80% and a median PFS of 9.2 months (67).

Of note, ibrutinib carries a low but notable risk of invasive aspergillosis (generally <5%), especially in patients receiving concomitant corticosteroids. While corticosteroid minimization is recommended, the use of antifungal prophylaxis remains controversial due to potential drug interactions.

③ Immunomodulatory drugs: moderate efficacy.

Lenalidomide, alone or in combination with rituximab, has demonstrated encouraging activity in relapsed/refractory PCNSL. Phase 1 and 2 studies have reported ORRs of 64%–67% and median PFS durations of 6–8 months (83, 84). As a maintenance strategy, lenalidomide has shown potential in prolonging remission in the salvage setting (84). Pomalidomide, a third-generation immunomodulatory agent, combined with dexamethasone, yielded an ORR of 48% and a median PFS of 5.3 months in a phase 1 study of 29 patients (85).

Combination trials of lenalidomide and ibrutinib are underway in both relapsed/refractory and frontline induction settings. Given their limited efficacy as monotherapy and potential for synergism, these agents are best reserved for clinical trial settings when available.

④ CAR-T cell therapy: emerging frontier.

Chimeric antigen receptor T-cell (CAR-T) therapy targeting CD19 has emerged as a promising investigational modality in PCNSL. Early trials suggest feasibility and manageable safety profiles. In a phase 1/2 study of tisagenlecleucel in 12 patients with recurrent PCNSL, the treatment was well tolerated, with only one grade 3 immune effector cell-associated neurotoxicity syndrome (ICANS) event and no treatment-related deaths. The ORR reached 58%, although all patients received bridging therapy (86). Importantly, in February 2026, the U.S. FDA updated the YESCARTA (axicabtagene ciloleucel) prescribing information, removing the prior Limitation of Use for primary central nervous system lymphoma and incorporating data from the new clinical study in relapsed/refractory PCNSL (87). This regulatory update substantially increases the clinical relevance of commercial axi-cel in this setting. Nevertheless, careful patient selection and close monitoring for neurologic toxicity remain essential.

Radiotherapy in relapsed or refractory PCNSL: salvage, support, and strategic delays.

As in systemic lymphomas, PCNSL exhibits marked radiosensitivity. Radiation therapy (RT) remains an effective modality for immediate cytoreduction, offering rapid symptom relief and radiographic responses. However, unlike peripheral disease, PCNSL behaves as a diffusely infiltrative malignancy within the brain, often involving microscopic, radiologically occult foci. As such, focal irradiation is rarely curative, and definitive radiotherapy demands whole-brain coverage to address potential subclinical disease.

WBRT is often employed in the salvage setting when systemic options have been exhausted or rapid neurologic deterioration precludes further delay. Retrospective studies report radiographic response rates of 74%–79% in relapsed or refractory PCNSL, with a median OS of approximately 10–16 months following WBRT (88, 89). However, the benefits are tempered by significant risks of neurotoxicity, including cognitive decline, particularly in older adults or those with prior chemotherapy exposure.

For select patients with isolated lesions and preserved performance status, focal radiotherapy—either conventional or stereotactic—may be used as a temporizing measure. These approaches can provide short-term symptom control or cytoreduction but are not sufficient as monotherapy. Systemic therapy remains essential for addressing microscopic disease and preventing recurrence.

## 10 Long-term outcomes and real-world prognosis

PCNSL retains a degree of chemosensitivity that permits long-term disease control in a subset of patients. High-dose methotrexate-based induction therapy achieves overall response rates of 70%–80%, with CR in approximately 50% of cases (5). Durable remissions extending beyond a decade have been reported in select patients (90), though disease trajectories remain heterogeneous. Approximately 25% of patients exhibit primary refractory disease, and even among initial responders, relapses are common—often confined to the central nervous system.

Age and performance status remain the most robust prognostic factors, heavily influencing outcomes (see Prognostic Assessment above). Despite therapeutic advances, real-world data highlight the continued challenge of PCNSL management. Population-based registries, including the Surveillance, Epidemiology, and End Results (SEER) program and the Central Brain Tumor Registry of the United States (CBTRUS), report a gradual improvement in median OS—from 12 months in the 1970s to 26 months in the 2010s (91). However, among patients aged ≥70, median OS remains dismal at just 7 months, underscoring the disproportionate burden in elderly populations.

In the relapsed or refractory setting, prognosis remains poor. Without salvage therapy, median OS is approximately 2 months; with treatment, it modestly improves to 7 months (8, 91). These sobering figures reinforce the urgency for novel strategies and individualized care pathways to extend survival and preserve neurologic function.

## 11 Summary treatment pathway for primary CNS lymphoma

**Figure 1** provides an at-a-glance visual roadmap of modern management in PCNSL. It places baseline prognostic assessment (age, performance status, deep-site involvement) alongside the now-established sequence of high-dose methotrexate-based induction, response evaluation, and risk-adapted consolidation—favoring thiotepa-conditioned autologous stem-cell transplantation in fit younger patients, and non-myeloablative chemotherapy or reduced-dose whole-brain radiotherapy in those with limited tolerance—followed by long-term surveillance. The diagram also highlights how ocular or leptomeningeal involvement is integrated into the pathway, and points to emerging treatment frontiers, including BTK inhibitor- or IMiD-based regimens, CAR-T cell therapy, and biomarker-guided approaches (e.g., MYD88 and CD79B mutations). By condensing a complex therapeutic landscape into a single schematic, the figure serves as a concise “clinical snapshot” that complements the text, allowing readers to trace the logic of contemporary practice and future directions at a glance.

## 12 Conclusion

Therapeutic progress in PCNSL has been driven by the adoption of HD-MTX-based induction and the integration of risk-adapted consolidation with thiotepa-conditioned ASCT, reduced-dose WBRT, or non-myeloablative chemotherapy. These advances have transformed a once uniformly fatal disease into one in which long-term remission is achievable for a substantial proportion of patients. Nonetheless, outcomes remain suboptimal for older adults, those with comorbidities or impaired performance status, and patients with relapsed or refractory disease. Recent insights into genomic drivers of PCNSL, such as MYD88 and CD79B alterations, together with real-world evidence on induction–consolidation sequencing (e.g., MTR plus EA, Dave et al., 2025), have refined risk stratification and informed therapeutic decision-making. However, relapse after initial response continues to limit survival, and neurocognitive sequelae of both chemotherapy and radiotherapy underscore the need for less-toxic regimens. Future progress will hinge on integrating molecular biomarkers for patient selection, developing novel targeted and immune-based strategies—including BTK inhibitor- or IMiD-based combinations, CAR-T cell therapy, and rational maintenance approaches—and embedding neurocognitive endpoints into prospective trials. The challenge now is to translate these innovations into durable, well-tolerated, and broadly accessible treatment pathways that improve both survival and quality of life for patients across the age and fitness spectrum.
